# Supplementary material for: Rethinking HIV care for youth: Insights from qualitative research with youth in Chad
Source: PLoS One. 2025 Jun 24;20(6):e0309497. doi: 10.1371/journal.pone.0309497 (PMC12186976; doi:10.1371/journal.pone.0309497)
Supplement: S2 File — (DOCX) [file pone.0309497.s002.docx]

**S2 File: Flowchart of sub-divided participants in each group.**

***HIV negative group: male (N = 16)***


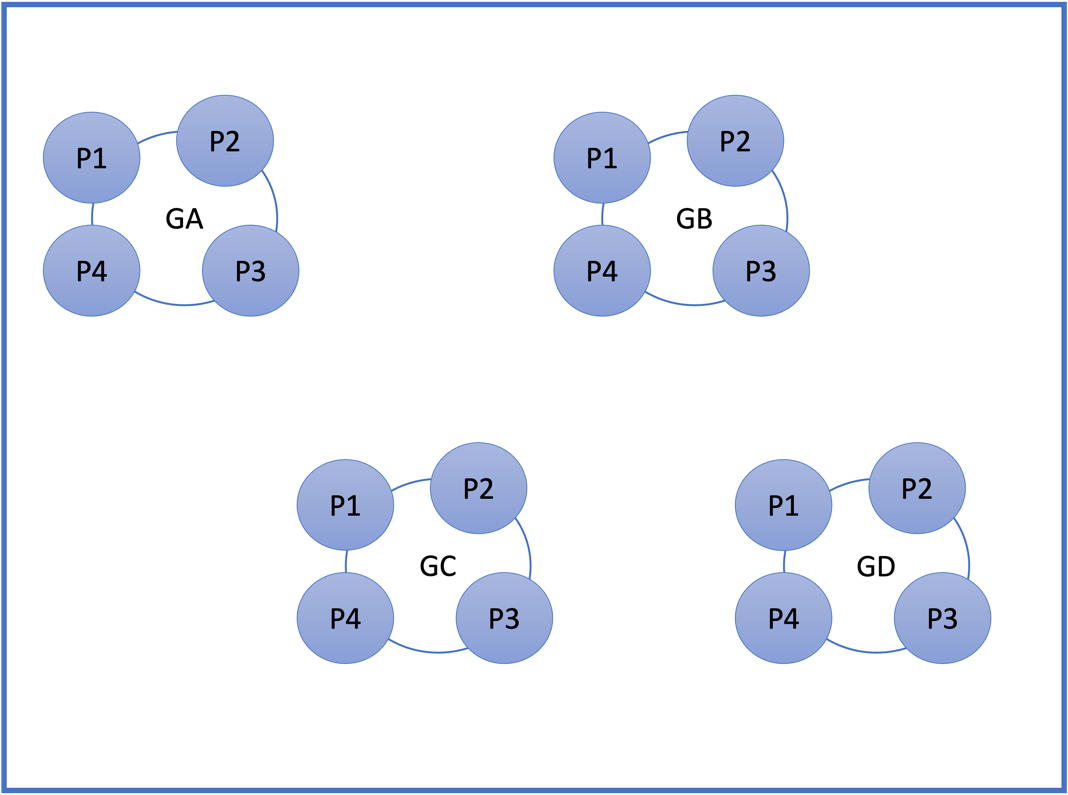


***HIV negative group: female (N = 13)***

**
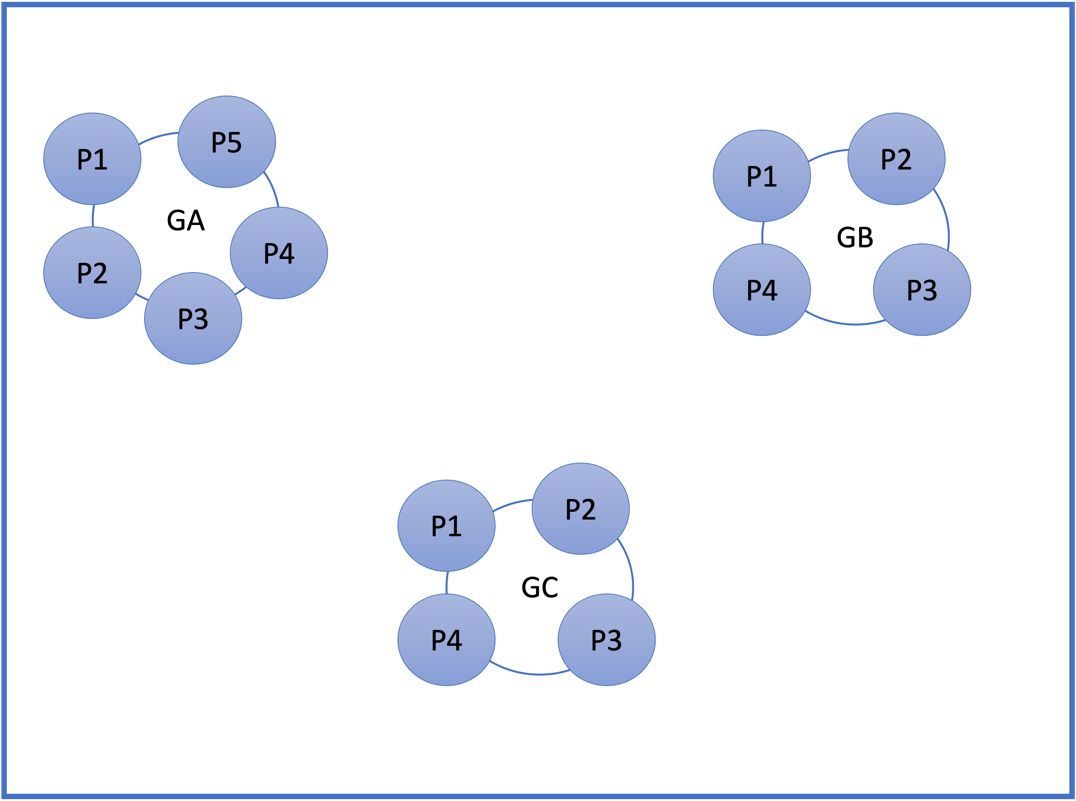
**

***HIV positive group: male (N = 12)***

***
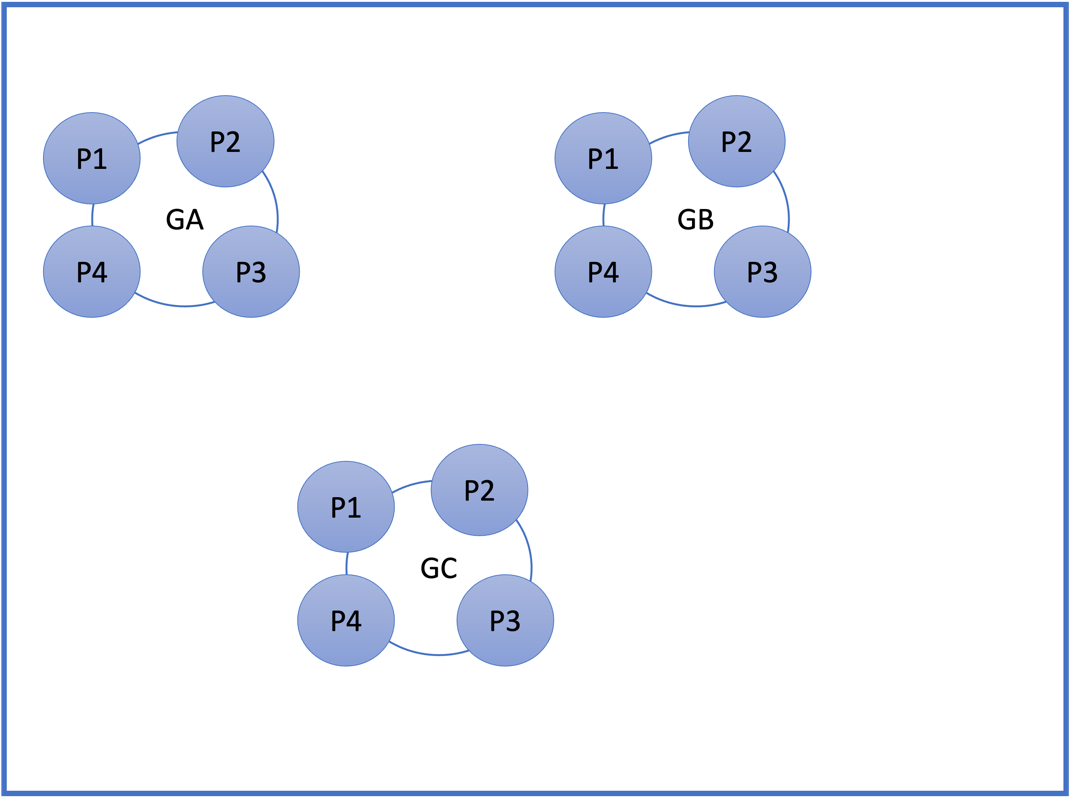
***

***HIV positive group: female (N = 11)***

**
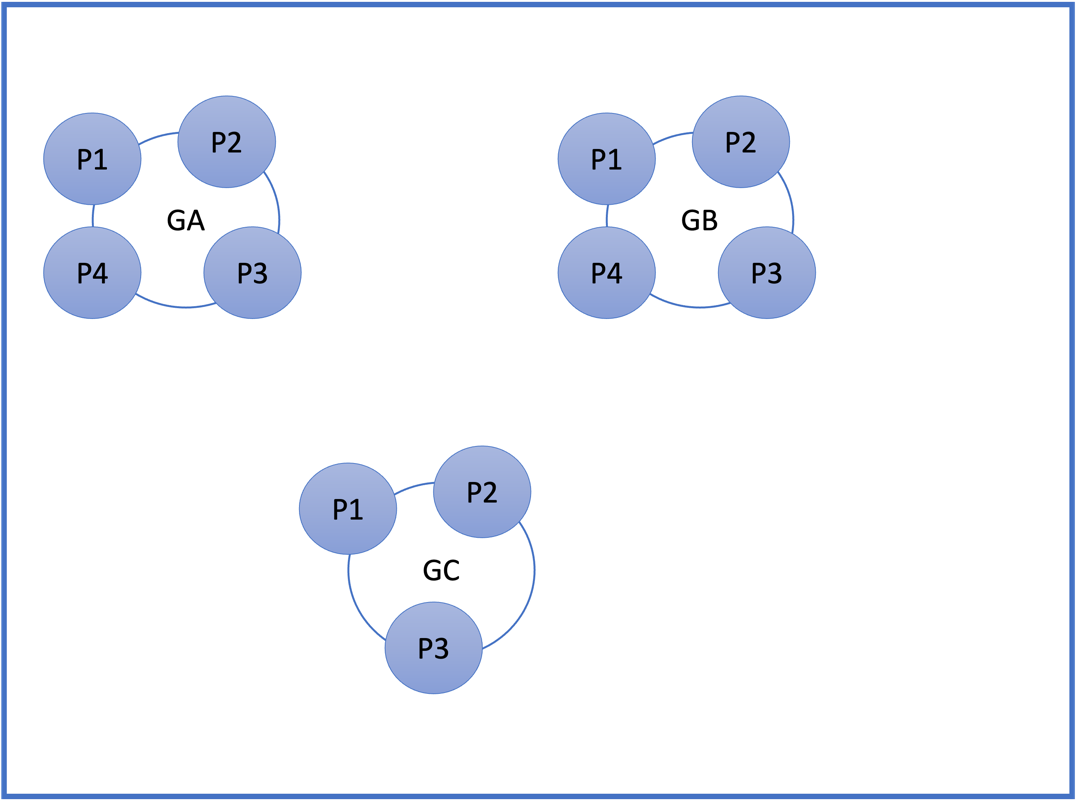
**
