# Supplementary material for: Rethinking HIV care for youth: Insights from qualitative research with youth in Chad
Source: PLoS One. 2025 Jun 24;20(6):e0309497. doi: 10.1371/journal.pone.0309497 (PMC12186976; doi:10.1371/journal.pone.0309497)
Supplement: Attachment — Submitted filename: DC_Reviewer_comments.docx [file pone.0309497.s010.docx]

Reviewer’s comments

Title: Rethinking HIV care for youth: Insights from qualitative research with youth in Chad.

Abstract

The introduction presents the problem and justification for seeking an in-depth understanding of the pathways to care for YLHIV. However, the study design is confusing. It is not clear which research methods that were adopted for the primary research versus those for the secondary analysis. It must be clear that the primary study design was Grounded Theory (GT). Also, GT is not a data analysis method but an inductive reasoning designed to generate theory. It is therefore not appropriate to suggest that GT design could be used to identify barriers and facilitators.

Line 26: In the presentation of results, the author should avoid words like ‘significant’ that cannot be supported by qualitative data and may be read as an exaggeration. Only data from youth is presented. There is no mention of the service providers.

Line 30 ‘unique’: How did your qualitative research data demonstrate that these challenges were unique to youth?

Line 31 ‘must be redesigned’: rephrase and present as a recommendation.

Introduction

Line 63: The study aim needs to be rephrased to reflect the qualitative nature of the research. Qualitatively you explore participants' experiences and views of the pathway.

Sampling

Line 75: In the context of this study, what defined ‘diverse’ and ‘relevant’?

Line 82: Why and how did you do a random selection from the list?

Line 84-94: Revise the paragraph. I assume the service providers were divided into three categories, not FGDs.

Data collection

Line 99-112: Details about the study sample that were expected under the sampling heading are found here. Consider carefully which information to present under the sampling heading versus what is relevant for the data collection section.

Data analysis

Line 140: ‘generating initial codes’. How did you incorporate the deductive codes, mentioned above, in this process?

Line 142: What were the ‘research questions’ for the secondary analysis?

Line 163: How was participant verification done?

Line 184: ‘Guaranteed’ is a big claim that cannot be supported.

Results

Line 207 Study characteristics: what does the author mean by this?

A brief description is recommended to introduce this section so that the reader can contextualize the different aspects presented here.

Line 208, the author starts by stating that Table 1 presents… while the expectation is for the author to present a description of the research findings and reference the Table.

Lines 208-212: Earlier on the author stated: “Transferability was facilitated by providing **thick descriptions** of the study context, participant demographics.” The presented demographics do not qualify as a ‘thick description’. I also could not find Table 1.

Line 246: Does this fall under the “Study characteristics” heading? If not I propose that the author creates headings that help the reader better contextualize the findings. Rather than presenting ‘Theme 1’ or sub-theme 1, I recommend that you remove this style of labelling. I also suggest that the author should reflect on the headings where possible rephrase and present in a manner that reflects the qualitative nature of the findings. But also headings could be used to reflect different aspects of the story that you are presenting.

Discussion

Line 470: “Findings from this study highlight the significant barriers and facilitators in the HIV care continuum for youth in Chad.” The statement is making a claim that is beyond the qualitative research. It is recommended that the author rephrase and present the discussion point in a manner that is reflective of the source of data and that the findings are based on the experiences and perceptions of the participants.

Lines 471-474: Reference the source for the statement beginning with: “*The ideal pathway to SRH…”*

The author tends to present findings in a quantitative language not supported by the qualitative research approach used in this study. I highlight this in the examples below:

Line 477: "Adolescent females facing higher barriers to accessing HIV testing due to societal norms and stigma." The statement implies a comparison and measurement of barriers faced by different genders.

Line 478: "Adolescent males faced barriers related to the convenience of testing facilities and the fear of receiving a positive status." This statement categorizes and quantifies the types of barriers faced by males.

Line 479: "Stigma was also identified as a main barrier to care and treatment adherence among youth living with HIV in many settings in sub-Saharan Africa [4, 34-36]." The use of "main barrier" suggests a quantitative assessment.

Line 484: "Financial and logistical barriers significantly hindered youth’s access to HIV testing and care services." The term "significantly hindered" suggests a measurable impact.

Conclusion

The conclusion should first reflect the research findings in line with the main study aim. At the moment the statement sounds like the author’s opinion. The logical flow from the study findings to the recommendation should be clear.
