# Supplementary material for: Rethinking HIV care for youth: Insights from qualitative research with youth in Chad
Source: PLoS One. 2025 Jun 24;20(6):e0309497. doi: 10.1371/journal.pone.0309497 (PMC12186976; doi:10.1371/journal.pone.0309497)
Supplement: Attachment — Submitted filename: Response_Review.docx [file pone.0309497.s012.docx]

Dr. Sara Jewett Nieuwoudt

Academic Editor

PLOS ONE

December 24^th^, 2024

Dear Dr. Sara Jewett Nieuwoudt,

Thank you for your insightful and constructive comments regarding our manuscript. We have taken a great consideration for each of the comments provided. We provide a detailed response to each of the points raised:

1. Editor’s comments
   a. Reviewed and updated the labelling of my supplementary files within the text.
   b. Provided additional clarification on who was involved in the secondary data analysis.
   c. Removed periods after the headings for tables and figures.
   d. Placed Tables within the body of the manuscript directly after the paragraph in which it is first cited.
2. Journal requirements
   a. Ensured that the manuscript meets PLOS ONE’s style requirements, including those for file naming.
   b. Created a new section called “Funding” to state the financial disclosure, and the role the funders took in the study.
   c. Amended the Role of Funder statement within my cover letter.
   d. Updated the Data Availability statement.
   e. Included captions for my Supporting Information files at the end of my manuscript and updated in-text citations to match accordingly.
3. Reviewer’s comments
   a. Abstract
      i. Clarified the study design to specify that the study is a secondary data analysis based on a parent study that employed a Grounded Theory design to develop theory inductively.
      ii. Removed the term “significant” to avoid exaggeration as it cannot be supported by qualitative data.
      iii. Added findings from healthcare workers (service providers), emphasizing their perspectives on barriers, facilitators, and their roles in the HIV care continuum.
      iv. Rephrased the statement to make it a clear recommendation rather than a prescriptive assertion.
   b. Introduction
      i. Rephrased the study aim to reflect the qualitative nature of the research.
   c. Sampling
      i. Defined “diverse” and “relevant” in the context of this study.
      ii. Addressed the random selection question for the selection of participants.
      iii. Revised the paragraph to mention three categories instead of focus group discussions.
      iv. Re-wrote all numbers from one to ten from numbers to letters.
   d. Data collection
      i. Moved all details about the recruitment criteria, planned participant numbers, and random selection process to Sampling.
      ii. Clarified definitions of diversity and relevance in Sampling.
      iii. Organized details about service provider categories and facility eligibility under Sampling.
      iv. Focused on the process and logistics of conducting FGDs.
      v. Highlighted participatory activities and their role in data enrichment.
      vi. Kept descriptions of audio recording, transcription, and quality assurance in this section.
   e. Data analysis
      i. Clarified how deductive codes were used as a framework in the initial stages of coding and complemented by inductive coding to capture emergent themes.
      ii. Explicitly stated the research aims for the secondary data analysis.
      iii. Added a description of how participant feedback was sought and used to validate findings.
      iv. Replaced the word “guaranteed” with a more accurate description of efforts to ensure reliability and cultural accuracy.
      v. Retained the explanation of working with original languages while empathizing the rationale behind this approach.
   f. Results
      i. Added an introductory paragraph to contextualize the findings.
      ii. Described demographics and referenced clearly.
      iii. Expanded on participant demographics and their relevance to study findings.
      iv. Created descriptive headings reflecting the qualitative nature of the findings, removing numerical labeling for subthemes.
      v. Ensured findings were described before referencing tables and figures, per reviewer feedback.
   g. Discussion
      i. Rephrased statements to reflect that findings are based on participants’ experiences and perceptions, avoiding generalizations not supported by qualitative data.
      ii. Rephrased statements that implied comparison, measurement, or quantitative assessments.
   h. Conclusion
      i. Rephrased conclusion to link findings to the aim.
      ii. Avoided opinion-like statements, grounding conclusions in the data.
      iii. Maintained a focus on actionable recommendations while emphasizing the study’s contribution to knowledge.

We are confident that these revisions have strengthened our manuscript and addressed the concerns raised. We hope that the revised manuscript will now be considered favorably for publication at PLOS ONE.

Thank you once again for your constructive feedback. We are available to address any queries or provide additional information as needed. Please address all correspondence to: [esias_bedingar@g.harvard.edu](mailto:esias_bedingar@g.harvard.edu) or [esias.bedingar@gmail.com](mailto:esias.bedingar@gmail.com).

Sincerely,

Esias Bedingar, SM, PhD on behalf of the author team.
