# Supplementary material for: Rethinking HIV care for youth: Insights from qualitative research with youth in Chad
Source: PLoS One. 2025 Jun 24;20(6):e0309497. doi: 10.1371/journal.pone.0309497 (PMC12186976; doi:10.1371/journal.pone.0309497)
Supplement: Attachment — Submitted filename: Response_Review_auresp_2.docx [file pone.0309497.s013.docx]

Dr. Sara Jewett Nieuwoudt

Academic Editor

PLOS ONE

May 3^rd^, 2025

Dear Dr. Sara Jewett Nieuwoudt,

Thank you again for your constructive and thoughtful comments regarding our manuscript. We have carefully reviewed and addressed all feedback from both the editorial team and the reviewer. Below, we provide a point-by-point summary of the changes made to the manuscript, including a response to the most recent request regarding references accuracy and completeness.

1. Editor’s comments
   a. Made a final round of revisions based on the feedback of the second reviewer.
2. Journal requirements
   a. Added additional references and edited the reference list. The references are #34 (lines 824-826), #40 (lines 843-845), #51 (lines 882-884), #52 (lines 885-887), #53 (lines 888-890), and #54 (lines 891-893).
3. Reviewer #2 comments
   a. Introduction
      i. Added a section on the legal and policy-related obstacles to accessing HIV services.
   b. Methods
      i. Addressed comment #2 by adding more information on the stratification of FGDs by HIV status in the Ethical approval section.
   c. Discussion
      i. Addressed comment #4 by reviewing and updating our reference list to ensure it is complete and accurate.
   d. Minor suggestion
      i. Changed the term “adolescents” to “youth” and “young people” when speaking to responsive care.
      ii. Removed the acronym “LGBTQ+” from the acronym list.

We are confident that these revisions have substantially strengthened our manuscript and fully addressed the reviewer and editorial feedback. We respectfully resubmit our revised manuscript and hope it will be considered favorably for publication in *PLOS ONE.* Please do not hesitate to contact us with any further questions.

Warm regards,

Esias Bedingar, SM, PhD on behalf of the author team.
