# Supplementary material for: Rethinking HIV care for youth: Insights from qualitative research with youth in Chad
Source: PLoS One. 2025 Jun 24;20(6):e0309497. doi: 10.1371/journal.pone.0309497 (PMC12186976; doi:10.1371/journal.pone.0309497)
Supplement: Attachment — Submitted filename: Response to Reviewers.docx [file pone.0309497.s014.docx]

Dr. Sara Jewett Nieuwoudt

Academic Editor

PLOS ONE

June 1^st^, 2025

Dear Dr. Sara Jewett Nieuwoudt,

Thank you again for your constructive and thoughtful comments regarding our manuscript. We have carefully reviewed and addressed all feedback from both the editorial team and the reviewer. Below, we provide a point-by-point summary of the changes made to the manuscript, including a response to the most recent request regarding references accuracy and completeness.

1. Editor’s comments
   a. Made a final round of revisions based on the feedback of the second reviewer.
   b. There is no explicit use of the word “adolescent” in the abstract, and the abstract already aligns with the youth-friendly framing, consistently using the term “youth”.
2. Reviewer #2 comments
   a. Minor suggestion
      i. Although it was already explicit through the quotes used on page 18, I edited the paragraph to make it more explicit.

We are confident that these revisions have substantially strengthened our manuscript and fully addressed the reviewer and editorial feedback. We respectfully resubmit our revised manuscript and hope it will be considered favorably for publication in *PLOS ONE.* Please do not hesitate to contact us with any further questions.

Warm regards,

Esias Bedingar, SM, PhD on behalf of the author team.
